# Excellent Fire Retardant Properties of CNF/VMT Based LBL Coatings Deposited on Polypropylene and Wood-Ply

**DOI:** 10.3390/polym13020303

**Published:** 2021-01-19

**Authors:** Zeeshan Ur Rehman, Atif Khan Niaz, Jung-Il Song, Bon Heun Koo

**Affiliations:** College of Mechatronic Engineering, Changwon National University, Changwon, Gyeongsangnam-do 51140, Korea; Zeeshan.physics@gmail.com (Z.U.R.); atif_khan_niaz@yahoo.com (A.K.N.); jisong@changwon.ac.kr (J.-I.S.)

**Keywords:** cellulose nano-fibrils, fire retardant coatings, DLVO theory, FTIR, UL-94, LBL

## Abstract

In this report, layer by layer (LBL) fire retardant coatings were produced on wood ply and Polypropylene Homopolymer/Flax fiber composites. FE-SEM and EDAX analysis was carried out to analyze the surface morphology, thickness, growth rate and elemental composition of the samples. Coatings with a high degree of uniformity were formed on Polypropylene composite (PP/flax), while coatings with highest thickness were obtained on wood ply (wood). FTIR and Raman spectroscopy were further used for the molecular identifications of the coatings, which confirmed the maximum deposition of the solution components on the wood substrate. A physiochemical analysis and model was proposed to explain the forces of adhesion between the substrate and solution molecules. Fire protection and thermal properties were studied using TGA and UL-94 tests. It was explored, that the degradation of the coated substrates was highly protected by the coatings as follows: wood > PP/flax > PP. From the UL-94 test, it was further discovered that more than 83% of the coated wood substrate was protected from burning, compared to the 0% of the uncoated substrate. The flammability resistance of the samples was ranked as wood > PP/flax > PP.

## 1. Introduction

Every year, around the globe, fire causes massive loss to human life and property. The reason behind such tragic losses is the increasing use of polymers-based products, wood and other highly flammable goods. Although initiation of fire cannot be stopped due to the nature of the pyrolysis reaction, the speed of the fire, intensity of the fire and its flammability can be retarded using novel bulk and surface coating techniques, in order to provide sufficient time for human evacuation and emergency response measures [1].

Polypropylene and wood ply used in this study has widespread use in automotive, electrical, packaging, transport and commercial goods, chemical tanks and medical applications, due to its low cost, easy processing, low corrosion, excellent mechanical properties, durability, and fatigue properties [2,3,4]. However, the inherent flammability and melt dripping problems particularly with PP have restricted its widespread applications [5,6]. Previously, various efforts have been made to improve the fire vulnerability of the PP. For instance, when 25% wt% loading of pentaerythritol (PER) and ammonium polyphosphate (APP) were added to the PP matrix, a vertical burning test V-0 rating was achieved [6]. However, such high quantity loadings causes significant negative shift in the mechanical properties and migration of additives in the PP matrix. Furthermore, Zuo et al., incorporated poly (2-morpholinyl-4-pentaerythritol phosphate-1,3,5-triazine) PMPT, a novel intumescent fire retardant (IFR) into PP, achieving improved thermal stability and flame retardancy of PP with lesser effects on the mechanical properties [7]. Lai et al. introduced another IFR, poly(-ethanediamine1,3,5- triazine-o-bicyclic pentaerythritol phosphate) (PETBP) and achieved high durability and thermal stability [8]. However, precursors of these IFRs, such as cyanuric chloride [8,9] or phosphorus oxychloride [10,11] are highly reactive and toxic to the environment.

This situation created a strong demand for environmentally friendly materials and techniques to be adopted for fire protection purposes. In this regard, extracted deoxyribonucleic acid (DNA), starch, chitosan and nano-fibrillated cellulose (CNF) are among the novel materials to be considered for fire retardant research and applications [12,13,14,15]. Nano-fibrillated cellulose (CNF) as characterized from its nano-dimensions (3–15 nm in diameter and 0.7–3 µm in length) are derivative of cellulose, obtained from wood or other sources, [15,16] representing an interesting bio-based material due to its attractive properties. These properties include high specific strength and elastic modulus ~150 GPa combined with low weight [17], high specific surface area [18,19], low thermal expansion coefficient ~10^−7^ K^−1^ [20], recyclability and transparency [21]. Due to these interesting properties, CNF is used in several applications such as biomedical products [22,23], biodegradable packaging materials [24], transparent composites [25,26] and sensors [27,28,29,30]. However, the use of CNF as fire retardant coating materials is still nascent, limited by various constraints and specific to few kinds of substrates [31,32,33].

**Figure 1:**
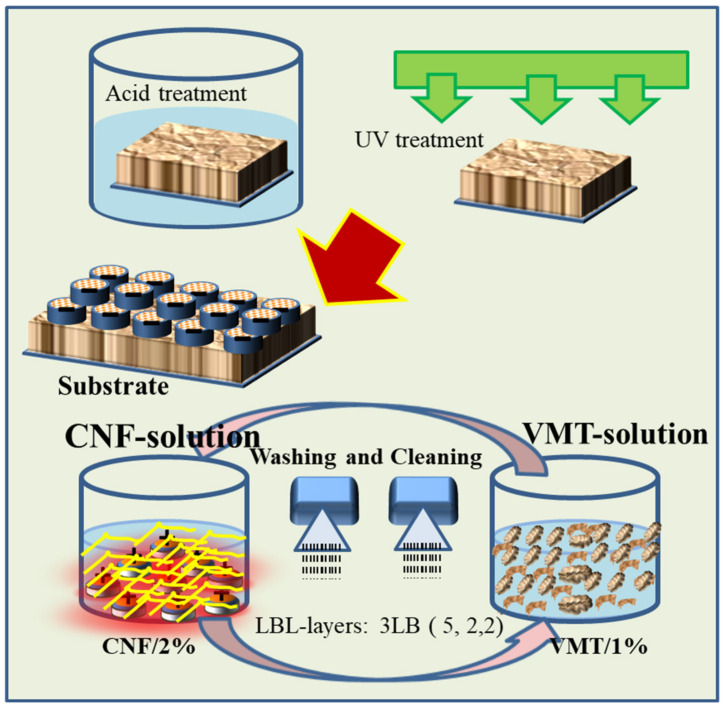
Schematic diagram of layer by layer (LBL) mechanism and process.

LBL is a process through which layer by layer deposition of various materials including CNF is carried out by dipping the substrate in alternate polyelectrolytes solutions. Chemical species in the polyelectrolyte solutions attach to the substrate by electrostatic attraction, yielding highly interpenetrated and uniform nanostructured coatings. Schematic presentation of the LBL process is shown in Figure 1. Reports suggest that fire protection properties of various substrates, such as cotton and polyester fabrics [34,35,36,37] polyamide and flexible polyurethane foams [38,39] have been highly improved using combination of LBL technique and appropriates materials. To the knowledge of the author, there is no generous literature related to LBL-based coatings on wood ply, polypropylene and its composite using a combination of cationized CNF and 2D VMT clay. Therefore, this project presents a novel contribution to produce nature-based VMT/C-CNF assembly for the first time on PP and wood ply substrate materials using LBL technique.

## 2. Experimental Section

### 2.1. Materials and Substrates

VMT clay (vermiculite) was obtained from a specialized local company. The obtained VMT was ground and sieved from a strainer of 50 µm mesh size. Cellulose nano-fibrils were obtained from ANPOLY, Gyeongju, Korea, prepared through TEMPO oxidized method. A semitransparent CNF gel was prepared using 2 wt% CNF fibers in deionized water (DI) with a conductivity ~1.4 µS/cm. The suspension was homogenized using long time stirring for about 48 h. The VMT solution was obtained using 1% of VMT platelets and was stirred for about 48 h.

### 2.2. Layer-by-Layer Assembly

LBL coatings were deposited on three kinds of substrates, i.e., wood ply (wood), molded Polypropylene Homopolymer (PP) and Polypropylene Homopolymer/Flax fiber composite (PP/flax). In order to activate the surface of the substrates, UV light and acidic solutions were used for initial substrate treatments. The substrates were initially soaked in 0.1 M HNO_3_ solution for 1 h. Subsequently, the substrates were dried and treated through UV light (80%) for 60 s. To deposit LBL coatings, the pre-treated substrates were alternatively immersed into CNF and VMT solution. The process mechanism and steps are shown in Figure 1. A total of 3 bilayers were deposited on each substrate. The first dipping process was maintained for 5 min and then dried at 60 °C while the next two bilayers were deposited by immersing the substrates in each solution for 2 min.

### 2.3. Measurements

The coated samples were analyzed through FTIR spectroscopy (JASCO 6300, Jeddah, Saudi Arabia), in the frequency range ~(4000 to 400 cm^−1^). The spectra profiles were recorded after 32 scans by FTIR spectrometer. Raman analysis was carried using Raman spectrometer (JACSCO, JP/NRS-3300). The laser beam wavelength used for the Raman analysis was ~785 nm. For FE-SEM, the substrates were coated with a Pt layer, and morphologies were observed using FE-SEM (TESCAN, Pleasanton, CA, USA) (CZ/MIRA I LMH). Elemental analysis of the overall coating surface and localized positions were carried out using EDAX connected with the FE-SEM. Transmission electron microscopic (TEM) images of the CNF nano-fibrils were obtained by Bio-TEM using low acceleration voltage of 200 V (Anpoly, Gyeongju, Korea).

### 2.4. Thermal Protection Properties

To analyze the sample for thermal degradation, TGA of samples were carried out under nitrogen atmosphere (20 mL/min) using Perkin Elmer Pyris-1 (Waltham, MA, USA) instrument in the temperature range (50 to 600 °C) at a heating rate of 20 °C/min. The flame test was carried out using UL-94 horizontal flammability test. Shape and dimensions of the specimens are shown in the discussion sections. Each specimen was exposed to the fire flame for 10 s, and the flame was then removed. Time and residue were recorded after the flame was removed.

## 3. Results and Discussion

### 3.1. Surface Morphology and Growth Analysis

**Figure 2:**
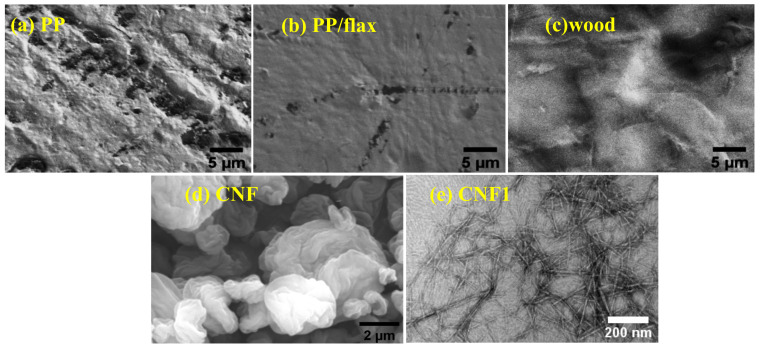
Surface morphology of the coatings formed on various substrates obtained through FE-SEM., (**a**) Polypropylene Homopolymer (PP) (**b**) PP/flax (**c**) wood (**d**) nano-fibrillated cellulose (CNF) (**e**) CNF1.

Surface morphology of the coating layers formed on various substrates can be seen in Figure 2a–c. It can be seen that surface of coatings on PP is porous, irregular and divided into portions and layers. Such irregular structure causes partial coverage of the substrate, which finally affects the protection properties of the coatings. These sub-portions and overlapping layers are composed of micro-granular structure. As the surface was charged using UV technique, it could be possible that the charge distributed preferentially on the surface, thus leading to the formation of inhomogeneous interaction domains and final layers. The surface charge “σ” accumulated on the substrates surface by the UV can be written using the Grahame equation [40]

**Figure 3:**
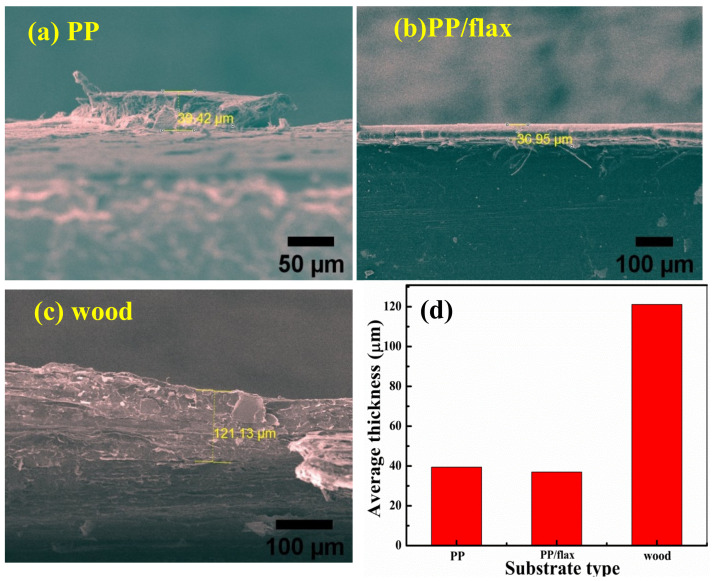
Cross section profiles of the coating through FE-SEM (**a**) PP (**b**) PP/flax (**c**) wood and (**d**) average thickness values.

(1)  σ=8kTϵϵon∞sinhzeψo2KT.

where k is Debye length, ψ_o_ is the surface potential. Furthermore, it can also be possible that localization of charges and thus the inhomogeneity could be caused by the steric and other effective adhesion forces. The major attraction responsible for adhesion between the solution components and substrate can be obtained using simple parallel plats approximation. Considering the CNF as 2D materials with infinite width and length compare to negligible thickness, as an extension to the work of Buning et al. [41], the double layer interaction can be written as

(2) $$V_{\mathrm{DL}}=64\mathrm{nkTx}{Ƴ}^{2}S\frac{1}{k^{2}}\exp\left(-\kappa H\right)$$

where S is the surface area that is equal to wL for parallel cubic prismatic rods having width denoted by w and length by L. The approximation is valid in this case as reported elsewhere [42]. Similarly, having the stated assumption, the van der Waals attraction can be fitted for flat-plan surface geometry as;

(3) $$V_{\mathrm{VW}}=-\left(\frac{A}{12{\pi H}^{2}}\right)S$$

where H’ is the distance between the the cellulose fiber and the substrate surface, and “A” is the Hamaker constant ~5 × 10^−21^ j [43]. In case of PP/flax, as in Figure 2b, it can be seen that the surface is highly uniform and densely packed. Furthermore, there can be seen very small size of VMT sheets distributed randomly on the surface, suggesting the effective LBL deposition. Surface uniformness could be attributed to the enhanced effect of UV activation and the negligible role of non-DLVO forces (bridging, solvation, steric and hydrophobic forces) [40,41,42,43,44]. It is important to mention that the presence of micro and nanofibers in the PP/flax matrix substrate could have played a significant role in the uniform activation of surface upon UV interaction. In the case of wood, the surface has compact layers structure, with minimum porosity and patches distribution. Due to the higher roughness of the wood substrate, the steric forces could also play a role in the interaction process between substrate and solution components. Thus, the coatings on wood have higher grain size and thickness compared to other coatings. During the LBL process, in addition to electrostatic and van der Waals interactions, friction, steric, capillary condensation and hydration forces could also contribute; however, compared to the strong effect of the former two forces, effects of the later forces can be neglected, or the attachment caused by these later forces could be sacked during the washing process. Figure 2d–e shows the cellulose nano-fibrils morphology and structure. At lower resolution, as obtained through FE-SEM, precipitate-type morphology appeared. However, further resolved through low voltage TEM, as obtained from Anpoly, the morphology composed of micron size fibrils. Growth characteristics were studied using thickness of the cross-section layers. Thickness of all the coatings deposited on the substrate can be seen in Figure 3d. High values of thickness can be observed in the case of wood coatings, while the other samples have relatively lower values of thickness. Deposition of the thicker layers on the wood can be attributed to the initial surface roughness (Amontons’ Law at micro scale) and the effective van der Waals interaction as wood ply have better surface charge accumulation.

Growth of the multilayers assembly follows the relation obtained experimentally from the fit of QCM negative frequency change [45].

(4) $$-Ɗf=A_{n}+\mathrm{Bexp}\left(R_{o}n\right)-1$$

where “n” is the number of single layer, A, B represents the amplitude of contribution with linear growth and exponential growth respectively. Surface uniformity and adhesion of the coatings can also be observed from the cross section, which confirm the highly uniform coatings in the case of PP/flax. Furthermore, the layers in the case of PP/flax are highly smooth and free from dendrites, broken parts or patches. This suggests excellent deposition of the layers on the PP/flax substrate.

### 3.2. Spectroscopic Analysis

**Figure 4:**
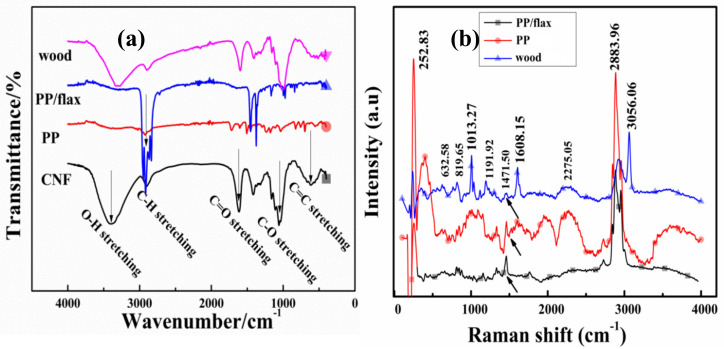
(**a**) FTIR and complementary and (**b**) Raman analysis for each coating.

Figure 4a shows the FTIR spectra of the individual CNF and coatings. A broad absorption band can be seen around at 3370–3250 cm^−1^, which can be attributed to the stretching vibrations of the OH groups attached to the cellulose structures in the CNF [40]. Such peaks can be observed with high intensity in the case of wood compared to other samples, which suggests maximum deposition of the coating material on the wood. In the middle wavelength ranges, the peak detected at 1614 cm^−1^ has two possible causes, i.e., bending mode of the absorbed water or stretching C=O bond [46,47]. Another peak can be traced at 1061 cm^−1^, which was caused by the asymmetric stretching of C-O-C bond in the cellulose. The stretching vibrations of the C–O–C bond, which are signified by β (1→4)-glyosidic linkage, occur as consequence glucose structure vibration and contribution from the OH bending [48]. In case of PP, the peaks are very weak and un-identifiable, suggesting lesser quantity of cellulose/VMT matrix deposited through LBL process. The FTIR results can be seen as in agreement with the literature [48,49,50].

For complementary observation and deposition features, Raman spectrums of the coatings were obtained as shown in Figure 4b. For homo-atomic bond, Raman is more effective than IR, as in the case of C=C bond [51]. Therefore, polar functional groups can be resolved effectively through strong IR bands, while vibrations of non-polar groups can be easily resolved through Raman lasers [52,53,54]. In Figure 4b, the peak at ~2883 cm^−1^ represents CH and CH_2_ stretching, while the peak at ~1608 cm^−1^ represents H–C–H and H–O–C bending. The peaks in the middle range at ~1471.5 cm^−1^ and ~1191 cm^−1^ signify H–C–C, H–C–O and H–O–C bending respectively. The lower range peaks obtained at ~1013 cm^−1^ represent C–C and C–O stretching, while the peak at ~819 cm^−1^ can be attributed to C–O–C in plan symmetric bending [55,56,57,58]. It can be observed that the Raman bands are fewer and lightly overlapped compared to that of infrared spectrum. In Figure 4, C–O stretching was observed in both IR spectrums ~1614 cm^−1^ as well as in Raman spectrum at~1608 cm^−1^. However, the major difference appears in the range ~ (200–500 cm^−1^), which is caused by the aromatic C=C stretching as shown Figure 4b. Peaks in the lower range of wavelength, specifically attributed to C=C stretching, can be seen with high intensity in the Raman profile compare to that in the IR profile, suggesting the proportional deposition of CNF/VMT coatings on the substrates. Peaks obtained from the coated PP/flax and wood were very sharp and clearly distinguishable, which suggest the high polycrystalline structure and well pattern layer by layer coatings.

**Figure 5:**
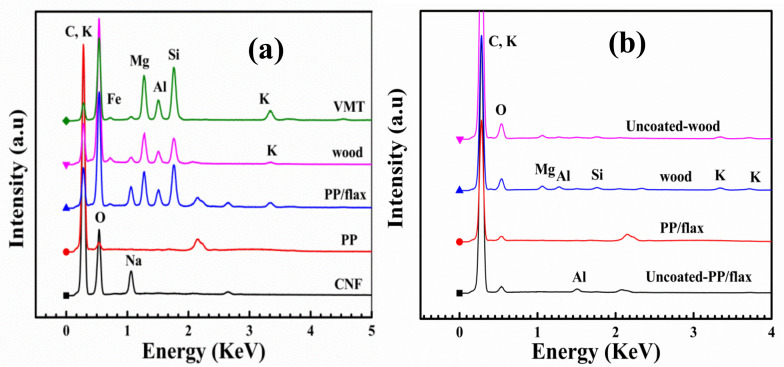
Elemental analysis spectroscopy of the coatings (**a**) before burn test and (**b**) after burn test.

In order to carry out compositional investigation of the coating layers, elemental analysis profile was obtained as shown in Figure 5a. It can be seen that “Na” and Al, Si, K, Mg are particular peaks of VMT sheets. All coated specimens, except PP, revealed traces of the elements from VMT in a dominant quantity ~50% as mentioned in Table 1. Similarly, the traces of CNF composition can be confirmed in the coating profiles. Furthermore, the quantity of “C” increased in the coating profiles compare to the individual entities.

### 3.3. Thermal and Fire Resistance Properties

**Figure 6:**
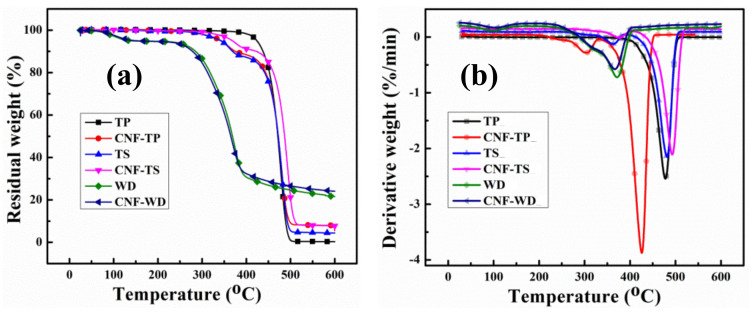
TG and DTG profiles of the non-coated and coated substrate under nitrogen atmosphere.

Thermogravimetric (TGA) analysis was carried out to observe the thermal degradation behavior of each coated substrate as shown in Figure 6, (a) weight loss curve and (b) derivative curves. The residue amounts at 300, 500 and 600 °C for each substrate are summarized in Table 2. 

It is evident from the derivative-curves, that there is significant increase in the degradation temperature for all the coated samples, which suggest enhanced thermal protection. Similarly, the residues obtained for the coated samples were significantly higher compare to the uncoated samples. The degradation of the uncoated wood occurred at around ~350 °C, which is caused by two competitive pathways: the depolymerization of glycosylic units into volatile products (levoglucosan) and the decomposition of the same units into thermally stable aromatic char (residue) as reported [59]. It is believed that the degradation of coated and uncoated wood followed the same route, except the significant decrease in degradation rate of the coated wood and final residue as shown in Table 2. At 600 °C, less than 21.4 wt% residue was obtained for uncoated wood. However, adding only 3BL, the residue weight increased up to ~24.5% which is 3 order of higher magnitude than the uncoated wood. The residual differences can also be seen at the pre-final stages (around 300 °C). The increase amount of residue for coated samples clearly demonstrates the protection of substrates during TGA test. The onset temperatures of PP/flax and PP are lower than the uncoated PP and PP/flax substrates, which can be attributed to the earlier degradation of the CNF-based layers that could possibly degraded earlier to form the intumescent char [60]. However, such intumescent char could shield the substrate from flaming; therefore, the lowered decomposition temperature is likely necessary rather than a defect of the CNF/VMT based LBL-layer system. The protection of the intumescent char can be confirmed from the residual weight at higher temperature. In case of wood samples, the difference in the onset temperature cannot be observed evidently; however, the residue at higher temperature is higher for coated wood, compared to the uncoated wood substrates. Besides, a consistent reduction in residue % age at 350 °C can be seen for all the coated substrates, which verify the initial degradation of the CNF-VMT matrix layers, thus protecting the substrates in the ending regime.

**Figure 7:**
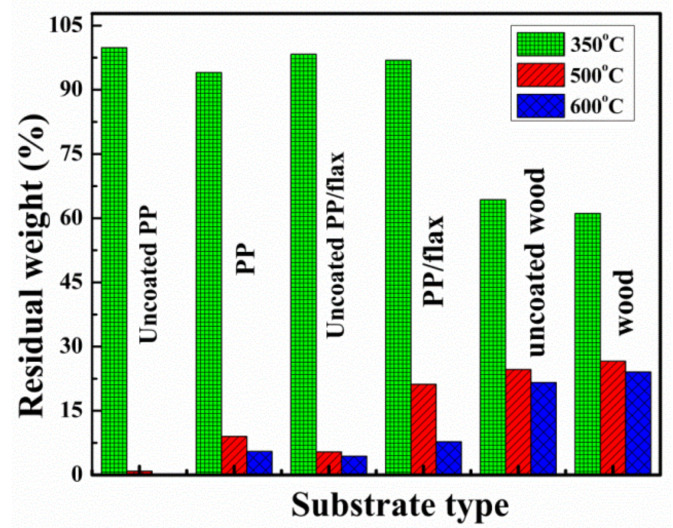
Comparative profile of the residue left at various temperatures during TG.

Comparison of the residual values at various stages for each coated and uncoated specimen can be seen in Figure 7. At higher temperature, the uncoated PP specimens completely vanished compare to its coated counter parts. In case of PP/flax, the residues left at 500 and 600 °C are quite high compared to those of uncoated PP/flax specimens, suggesting the effective resistance of the coating layers on PP/flax substrate.

**Figure 8:**
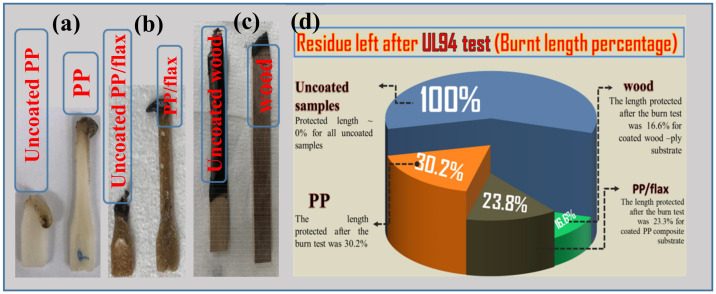
(**a**–**c**) Flame test specimens and (**d**) % of the residue left after flame test.

**Figure 9:**
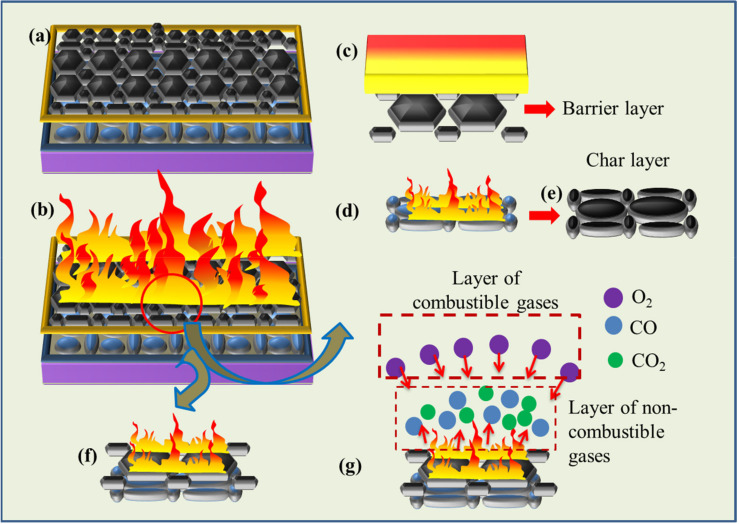
Threefold mechanisms offered by coatings to protect the substrates.

**Figure 10:**
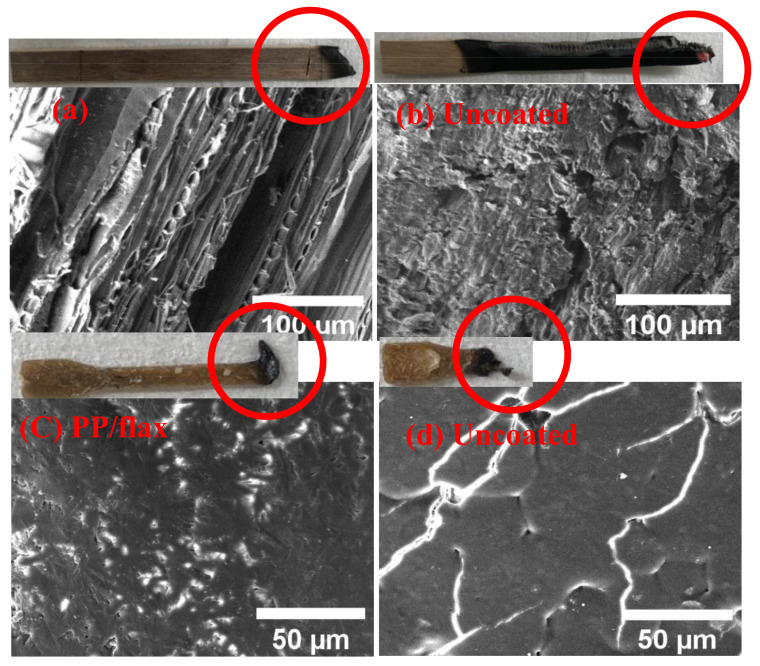
Surface morphology of the char produced after flame test. (**a**) coated wood, (**b**) uncoated wood (**c**), coated PP/flax and (**d**) uncoated PP/flax.

The same set of samples was put through UL-94 test (ASTM D6413). Flammability was tested by holding a butane torch on one side of the samples for 10 s. The flame heat causes stepwise effects, such as flame impingement, decomposition, ignition, flame spread and extinction processes. Ignition temperature, conductivity, density and specific heat of the deposited LBL coatings are highly responsible for retarding the fire. These are called thermal response parameters, which have direct relation with the ignition characteristics as given by the relations [61]:

(5)  tig=TRP q ′2

where “q’" is the net heat flux. During the flammability test, it was observed that a brighter and more vigorous flame was created after ignition for each uncoated substrate. This is well expected due to the high flamable nature of the uncaoted substrates. For each uncoated sample, no significant length (residue weight) was left. On the contrary, a significant residual length was left for each coated sample before extinguishing as can be seen in Figure 8a–c. The maximum protection of the substrate by the coatings was observed for wood and PP/flax, with leftover residue length as ~84% and 73% respectively. The enhanced thermal protection of the coated wood can be attributed to the higher thickness of the coating layers and in case of PP/flax, the improved adhesion between CNF and VMT resulting from the LBL structuring and uniformity [55]. Generally, three phenomena can be attributed to the fire protection mechanism offered by the coatings as shown in Figure 9a–g: (a) Excellent flame protection by the efficient thermal barrier by the coatings that causes reduction in thermal degradation of the substrate as also confirmed by thermogravimetric analysis (TGA). The schematic presentation of such barrier layer is shown in Figure 9c. (b) Protection by the char, produced as a result of CNF-VMT matrix. Since the coatings layers burns with time, the char produced by the CNF/VMT matrix act as a blocking agent to retard the fire access to the actual substrate as in Figure 9d,e. (c) Gases emission by the coating layers that cause saturation of the near-surface zone and thus limit the diffusion of oxygen to burn with carbon as in Figure 9f,g and prevent the chain reactions resulted from combustion as reported [1]. Morphological analysis of the char produced after the UL-94 test was carried out as shown in Figure 10a–d. PP/flax and wood char were selected for the after-burn FE-SEM analysis. It can be seen that char of the coated wood has regular and well-shaped cellular structure. Apparently, the coatings effectively retarded the fire to protect the structure from damage. However, the uncoated wood has broken sub-structures, due to the fatal damage caused by the fire. The cracks and broken parts have been used for the intake of oxygen to react the combustible gases, produced through degradation. It can be further confirmed from the EDS spectra, that the coated wood has significant percentage of solution components, i.e., (Mg, Al, Si) as mentioned in Table 3, which suggests the resistance offered by the coatings to large extent before degradation. Similarly, char of the coated PP/flax and uncoated PP/flax can be seen in Figure 10c,d. Coated specimen have compact structure with no cracks or broken patches; however, the uncoated counterpart can be seen as ruptured with larger cracks and patches. It suggests that uncoated PP/flax could not withstand against the fire, while 3 BLs coatings on PP/flax effectively protected the substrate from the significant mechanical damages. Therefore, addition of few BLs coatings, caused not only fire protection but also enhanced the mechanically stability of the substrates.

## 4. Conclusions

LBL coatings were successfully deposited on wood ply, PP and PP/flax substrates using CNF/VMT based polyelectrolyte solutions. From the surface analysis, relatively uniform layers were formed on PP/flax; however, rough and thick coatings were observed on the wood substrates. FTIR and Raman spectroscopy further confirmed the maximum deposition of solution components on the wood samples. Thermal degradation of the coated substrates suggested significant improvement in the residue weight at 600 °C as follows: wood (24.0%) > PP/flax (7.8%) > PP(5.5%), compared to the uncoated counterparts as 0.1%, 4.4% and 21.6%, respectively. Using UL-94 fire test, it was further explored that self- extinguishing was achieved with enhanced anti-flammability properties of the coated substrate as follows: wood > PP/flax > PP. The after-burn surface analysis suggested improvement in mechanical integrity of the original microstructure of the wood and PP/flax substrates. Three important mechanisms, i.e., physical barrier, char resistance and retardant gases emission, were suggested as being responsible for the improved fire-retardant properties of the wood and PP/flax substrates.

## Figures and Tables

**Table 1:** Elemental analysis before burn test.

Samples (At%)	C	O	Al	Na	Mg	K	Si
CNF	73.19	24.11		2.56			
PP	93.20	5.98		0.83			
PP/flax	39.85	45.83	1.71	1.14	4.4	0.54	3.7
wood	38.4	52.5	1.29	1.78	3.9	0.31	2.2
VMT	31.79	47.51	4.58		7.9	1.61	7.0

**Table 2:** Thermal parameters obtained from TGA analysis.

Samples	Residue (%) at 350 °C	Residue (%) at 500 °C	Residue (%) at 600 °C
Uncoated PP	99.83	0.88	0.1
PP	94.05	9.0	5.51
Uncoated PP/flax	98.3	5.4	4.4
PP/flax	96.96	21.2	7.76
Uncoated wood	64.3	24.64	21.59
wood	61.1	26.58	24.07

**Table 3:** Elemental analysis after-burn test.

Samples (At%)	C	O	Al	Au	Na	K
PP/flax	95.71	3.96	0.33	0.03		
Uncoated PP/flax	97.1	2.73		0.08		
wood	89.54	9.37	21.59	0.05	0.39	0.69
Uncoated wood	87.99	10.95		0.07		

## Data Availability

The data has been obtained from the experimental results.
